# Effect of lactylation on functional and structural properties of gluten

**DOI:** 10.3389/fnut.2022.1018456

**Published:** 2022-10-28

**Authors:** Yu Wang, Yan Li, Mingcong Fan, Li Wang, Haifeng Qian

**Affiliations:** State Key Laboratory of Food Science and Technology, School of Food Science and Technology, National Engineering Research Center for Functional Food, Jiangnan University, Wuxi, China

**Keywords:** lactylation, gluten, mechanism, functional property, structural property

## Abstract

Gluten is widely used as a high-quality protein material in the food industry, however, low solubility restricts its development and applications. In this study, gluten was treated with lactate and sodium lactate for lactylation. Lactylation of gluten altered surface charges of the protein, leading to a significant improvement in the solubility. An improvement in oil absorption capacity (OAC) could be attributed to a decrease in protein folding degree after lactylation. In addition, the emulsifying properties of gluten were significantly enhanced. The introduction of lactate group also significantly increased the viscoelasticity of gluten. Fourier transform infrared spectroscopy (FTIR) showed there was a significant decrease in β-turns content and a significant increase in β-sheets content. The folded conformation of gluten was gradually extended after lactation by fluorescence spectroscopy measurement. Both in lactate and sodium lactate treatment, the maximum emission wavelength indicated a blue shift, and the UV intensity showed an increase. These results could demonstrate that lactylation could extend the structure and improve the functional property.

## Introduction

Gluten, an abundant and cheap plant protein source, has been traditionally used in flours products (1). According to the different solubility, it can be divided into four categories: albumin, globulin, gliadin, and glutenin, among which the content of albumin and globulin is scarce, accounting for about 10% of the total wheat protein. The ratio of gliadin and glutenin is about 1:1, and the two proteins interact to form gluten network. Gliadin has monomer structures containing four groups including α (25%), β (30%), γ (30%), and ω-gliadins (15%) (2). The glutenin subunit has two fractions containing low-molecular-weight glutenin subunits (LMW-GS) and high-molecular-weight glutenin subunits (HMW-GS) (3). In food processing, the leading application is mainly reflected in two aspects. One is as a protein nutritional supplement. The other is like a dough reinforcing agent to improve the rheological properties of dough and product quality. Because gluten contains a lot of Gln, Pro, and hydrophobic amino acids bringing about its poor solubility, it is difficult to meet the needs of food industrial processing in practical application (4).

So far, a large number of modification strategies have been applied to improve the physicochemical attributes of gluten, including physical, chemical, and enzymatic approaches (5). Physical modifications change protein’s functional properties by changing aggregation state, spatial conformation, and the loose degree between peptide chains. Marcuzzo et al. (6) reported that ultrasound could improve gluten dispersion and hydrophilic surface properties. Chemical modification means that the functional groups of amino acid residues or peptide chains break, polymerize or introduce new groups through chemical reagent treatment, to change the static charge quantity, chemical forces inside the protein, relative molecular weight, amino acid composition, and surface hydrophobicity. It has been reported that polyphenols were an effective way to reduce the sensitization of gliadin and compared the efficiency of polyphenols from different sources to reduce the immunogenicity and allergenicity of gliadin (7). The functional properties of gluten were significantly improved and the internal structure was smoother after deamidation and succinylation (8). Besides, Wang et al. (9) effectively improved the rheological and thermal properties of gluten after glutamine transaminase and alkaline protease treatment. Within them, chemical modification has plenty of advantages compared with other treatment methods, including high efficiency, low cost, and good performance.

Lactate is the main end product of fermentation, which is produced by lactobacilli (10). Lactate is a food additive that is commonly used to apply in dairy products, meat products, and beverages, especially flour products. Lactate was more fit for modification on account of substantial reasons: First of all, lactate can be used as a sour agent, with a characteristic and mild acid taste. Next, during fermentation, organic acids, such as lactate, are often produced, which lower the pH of the dough (11, 12). Lactate inactivates protease by controlling the pH value so that increasing the cooking ability of flour products. Finally, it can effectively inhibit microbial growth and prolong shelf life. Meanwhile, it also exists in the blood and muscle tissue of mammals, which is converted from pyruvate. It has been regarded as a metabolic by-product for a long time, and its important regulatory role in biological functions has not been recognized (13). However, Zhang et al. (14) concluded that lactate accumulated during metabolism can act as a precursor resulting in lactylation of histone lysine residues, which directly stimulated gene transcription from chromatin. Hence, we speculate if there is lactylation that can change gluten’s conformation and structure and consequently its physicochemical and functional properties.

In the present study, a novel modification method of gluten is described. Lactylated gluten was obtained by treating with lactate and sodium lactate. Functional properties were analyzed through solubility, water holding capacity (WHC), OAC, emulsifying property, and rheological property. Furthermore, the structural characteristics of lactylated gluten were studied to determine the potential mechanism. All in all, our study aims to provide a new strategy for the modification of gluten and evaluate the effect of lactylation on gluten, which will broaden the gluten’s scope of application in industrial production.

## Materials and methods

### Materials and reagents

The commercial gluten was provided by Yihai Kerry (Jiangsu, China). The composition of gluten was measured according to the AACC International (2000, methods 46–13, 30–10, 76–13, 44–19, and 08–01) and the results were expressed as average values. The content of protein, fat, starch, moisture, and ash were 70.00, 0.73, 10.16, 8.40, and 0.40%, respectively. 5, 5′-dithiobis-(2-nitrobenzoic acid) (DTNB) was obtained from Shanghai yuan ye Bio-Technology Co. Ltd. Ethylene Diamine Tetraacetic Acid (EDTA) was purchased from Shanghai Hushi Chemical Co., Ltd. BCA protein assay kit was obtained from Beyotime (Shanghai, China). Sunflower seed oil was obtained from a local superstore. Pan anti-Kla (PTM-1401) antibodies were generated by PTM Bio Inc. All other chemicals and solvents were analytical grade.

### Treatments of gluten

The gluten sample was weighed (25 g) and mixed with 0.1 M lactate solution at a concentration of 10%. The reaction was carried out at room temperature for 3 h. Since the isoelectric point of gluten is about 7.0, after completion, the pH of the suspension liquid was adjusted to 7.0 with 2 M NaOH to precipitate the protein. The sample was then collected and freeze-dried for further analysis.

Part of the gluten was weighed and dissolved in 250 mL of deionized water (pH = 10.0) with a mass fraction of 10%, followed by adding 5 g of sodium lactate. The suspension was continuously stirred for 3 h at room temperature. During the whole reaction, the pH was adjusted to 10.0 with 2 M NaOH. Similarly, the pH of the solution was adjusted to the isoelectric point to ensure that the gluten was subsided. After completion of all reaction steps, the sample was frozen at –80°C and lyophilized for further analysis.

### Western blot

Protein samples were resolved on 10% sodium dodecyl sulfate polyacrylamide gel electrophoresis (SDS-PAGE) gels by using standard procedures and subsequently transferred onto PVDF membranes. Pan anti-Kla (PTM-1401) antibodies were devoted to western blot analysis.

### Solubility

The samples were dissolved in deionized water, and the pH of the mixture was adjusted to 3.0–10.0 with HCl and NaOH. The solution was agitated for 120 min at 25°C. The protein contents in the collected supernatant fractions were determined after centrifugation at 10,000 rpm for 15 min. The protein content of the supernatant was measured using a BCA protein assay kit.

### Water holding capacity and oil absorption capacity

First, the gluten sample (0.4 g) was weighed and put in a 15 mL centrifuge tube. After measuring the total weight (m_1_), 4 mL of water was added into the centrifuge tube to cover the sample and the sample was blended thoroughly with a vortex mixer. The blended sample was allowed to stabilize for 60 min before centrifugation at 4,000 rpm for 20 min. The supernatant was decanted and the total weight of the pellet and tube (m_2_) was measured.

A 0.3 g portion of the gluten sample was weighed and put in a centrifuge tube. Subsequently, the total weight (m_3_) was measured and the powder was covered by 2 mL of sunflower seed oil. The compound was mixed thoroughly and allowed to stand for 1 h. After completion, the sample was centrifuged at 4,000 rpm for 20 min. The supernatant was emptied and the total weight of the sediment and tube (m_4_) was measured.

The WHC and OAC were calculated as follows:


(1) $$\mathrm{WHC}\,\left(g/g\right)=\frac{{\text{m}}_{2}-{\text{m}}_{1}}{{\text{m}}_{0}}$$

(2) $$\mathrm{OAC}\,\left(g/g\right)=\frac{{\text{m}}_{4}-{\text{m}}_{3}}{{\text{m}}_{0}}$$

where the m_0_ represents the specific weight of the sample.

### Emulsifying activity index and emulsion stability index

Two criteria of emulsifying properties were measured: emulsifying activity index (EAI) and emulsion stability index (ESI). First, 5 mL sunflower seed oil was blended with 20 mL 10 mg/mL gluten solution (0.05 M phosphate buffer). Then, the mixture was homogenized at 10,000 rpm for 1 min by the T25 Ultra Turrax Homogenizer (IKA, Germany). Subsequently, 50 μL of the homogenized emulsions at the bottom of the tube were mixed with 4.95 mL 0.1% sodium dodecyl sulfate (SDS). The absorbance of the solutions at 0 and 10 min after dilution were measured at 500 nm. EAI and ESI were calculated based on the following equations:


(3) EAI(m/2g)=n×2×(2.303⁢A500)×10-4φ⁢CL

where A_500_ is the absorbance of the diluted emulsions at 500 nm (0 min), n is dilution multiple, φ is the volume fraction of the dispersed phase (0.2), L is the light path length in meters (0.01 m), and C is the protein concentration before emulsion formation (g/mL).


(4) $$\mathrm{ESI}\,\left(\min\right)=\frac{\Delta \,\text{T}\times {\text{A}}_{0}}{{\text{A}}_{0}-A_{\text{t}}}$$

where A_0_ and A_*t*_ are the absorbance of the diluted emulsions at 0 and 10 min, respectively; and ΔT is 10 min.

### Rheological

Rheological analysis was conducted using a DHR-3 rheometer (TA Instrument, New Castle, USA) equipped with a 40-mm diameter plate geometry. The samples were mixed with deionized water and rested for 30 min. After the sample was loaded onto the rheometer, the excess sample was removed. The edges of each sample were coated with silicone oil to prevent evaporation of sample water during measurement. A frequency sweep experiment of 0.1–10 Hz was carried out at 25°C with a strain of 1% (within the linear viscoelastic region). The storage modulus and loss modulus were recorded.

### Fourier transform infrared spectroscopy

The gluten samples (1 mg) and KBr (100 mg) were weighed and mixed, then fully ground and pressed into a transparent slice. The samples were scanned by Fourier transform infrared spectroscopy (Nicolet US IS10, Thermo Fisher Scientific, Waltham, MA, USA) from 4,000 to 400 cm^–1^ at a resolution of 4 cm^–1^. The contents of secondary structures were demonstrated by using Fourier self-deconvolution and fitting with the Gaussian function by Peakfit v4.12 software.

### Intrinsic fluorescence spectra

The change in the tertiary structure of gluten was determined through endogenous fluorescence spectroscopy (15). All samples were dissolved in 0.05 M phosphate buffer (pH = 7.0) and modulated to the final concentrations of 0.4 mg/mL. All samples’ intrinsic fluorescence spectra were collected by a fluorescence spectrophotometer (F-7000, Hitachi, Tokyo, Japan) with an excitation wavelength of 280 nm, and an emission wavelength of 300–500 nm. The specific parameters were performed at 1,200 nm/min with a response time of 0.5 s, a PMT voltage of 600 V, and excitation and emission slits of 5 nm. All results were repeated three times to ensure accuracy.

### UV absorption spectrum

UV Absorption Spectrum also was employed to predict the conformational change of gluten. A certain amount of gluten was weighed and dissolved in 0.05 M phosphate buffer (pH = 7.0). After the reaction was agitated for 2 h at room temperature, the mixture was centrifuged at 10,000 rpm for 15 min. All samples’ supernatant was uniformly adjusted at 0.4 mg/mL using a BCA protein assay kit. The UV absorption spectrum was recorded by a UV spectrophotometer (UV-1800) with a scan range of 200–400 nm, an optical path of 1 cm, and a response time of 0.1 s. Each sample was repeated at least three times.

### Determination of sulfhydryl of gluten

Approximately 50 mg gluten sample was weighed into the centrifuge tube, which was augmented with 1 mL of Tris-glycine-EDTA buffer (10.4 g of Tris, 6.9 g of glycine, and 1.2 g of EDTA per liter, pH 8.0). Then we added 4.7 g guanidine hydrochloride and diluted it with buffer solution to 10 mL. 1 mL solution above, 4 mL urea and guanidine hydrochloride (8 M urea solution and 6 M guanidine hydrochloride solution), and 0.05 mL Ellman’s reagent (DTNB in Tris-glycine-EDTA buffer) were added, and the sample was left standing in the dark at room temperature for 30 min. The absorption of the mixture was measured at 412 nm against the blank (without the gluten). The sulfhydryl (SH) content was calculated as follows:


(5) $$\text{SH}\,\left(\mu \,M/g\right)=\frac{73.53\,{\text{A}}_{412}\times \text{D}}{\text{C}}$$

where 73.53 is derived from 10^6^/(1.36 × 10^4^), 1.36 × 10^4^ is the molar absorptivity and 10^6^ is for conversions from the molar basis to the μM/mL basis and from mg solids to g solids, A_412_ is the absorbance at 412 nm, D is the dilution factor, 5.02, 6.04, and 30.2 for SH in flour or gluten, milk, and egg white, respectively, and C is the sample concentration (mg/mL).

### Sodium dodecyl sulfate polyacrylamide gel electrophoresis

The sample solution (1 mg/mL) was added to the loading buffer [50 mM Tris–HCl, 0.1% bromophenol blue, 10% glycerol (v/v), 2% SDS (w/v), pH 6.8] and heated at 100°C for 10 min. After cooling, 10 μL of the sample was loaded onto the gel, and the test was performed at 120 V for 60 min. The gel was stained with Coomassie Brilliant Blue, and then the gel was destained overnight at 37°C with a mixture of 10% acetic acid and 5.5% ethanol.

### Statistical analysis

The results were reported as mean ± standard deviation (SD). Data processing was performed with SPSS software (version 26, IBM Co., USA). The one-way analysis of variance (ANOVA) was used to analyze the data. The significant level was set at 5%.

## Results

### The validation of gluten’s lactylation

**FIGURE 1:**
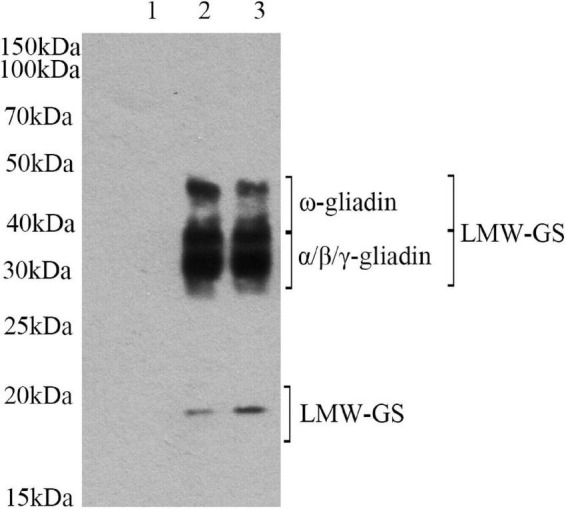
Western-blot analysis of gluten exposed to different treatments: (lane 1) raw gluten; (lane 2) gluten treated by lactate; and (lane 3) gluten treated by sodium lactate.

To determine if lactylation has occurred, we employed Pan anti-Kla antibodies for western blot analysis. Pan anti-Kla antibodies can recognize lactylation of amino acids and are important tools in the study of post-translational protein modifications. After PAGE separation, protein was transferred to the solid phase carrier. Solid phase carrier adsorbed protein in the form of non-covalent bond, keeping the same biological activities of protein. Solid phase carrier proteins, as antigen, conducted an immune response with the Pan anti-Kla antibody. Then it reacted with enzyme labeled second antibody. After ecl chemiluminescence method, it can be determined whether the target protein has been modified. The band of raw sample were different from that of lactylated samples (Figure 1). Lane 1 has no distinct band, which indicated that there is no lactylation in raw gluten. However, the band (15–20, 30–50 kDa) intensities of raw gluten samples were significantly strengthened after lactylation. The clear band at 30–50 kDa represented gliadin and LMW-GS, and the clear band at 15–20 kDa might be LMW-GS. These results confirmed the lactylation of gluten structure.

### Effects of lactylation on the functional properties of gluten

**FIGURE 2:**
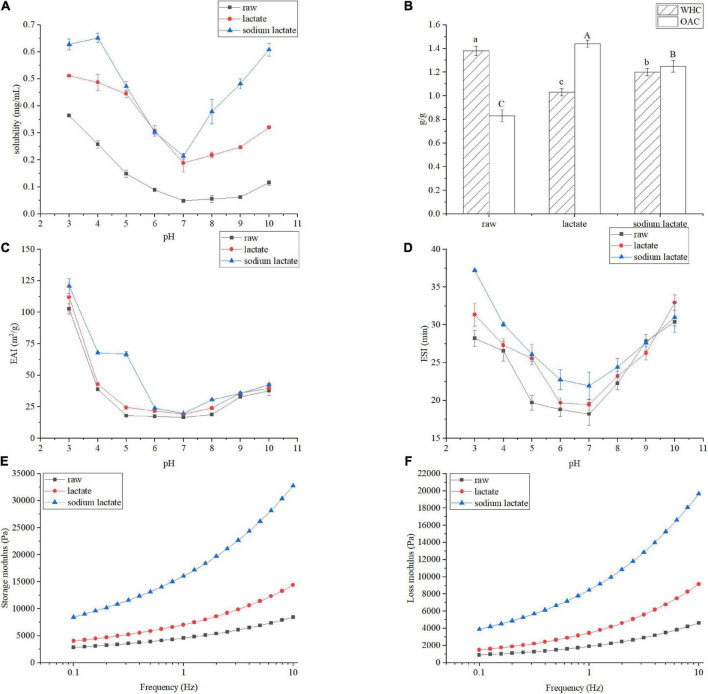
Changes in the functional properties of gluten sample. Solubility **(A)**, WHC and OAC **(B)**, EAI **(C)**, ESI **(D)**, storage modulus **(E)** and loss modulus **(F)**. All of the samples were tested in triplicate, and data are expressed as mean ± SD of three independent experiments for all panels.

Because functional properties of gluten were closely associated with their production and application, we first compared lactylation modified gluten samples with raw gluten, in terms of their functional properties. Figure 2A shows the solubility of the raw gluten and the gluten treated by lactate and sodium lactate at the pH range of 3.0–10.0. The solubility profile of all samples showed a U-shaped curve in which the protein solubility was the lowest near pH = 7.0. As pH is away from the isoelectric point, the solubility gradually increases. Gluten at pH 3.0 resulted in the highest solubility (0.36 mg/mL). The lactylation had a conspicuous effect on the solubility of gluten at pH values (3.0–10.0) (*p* < 0.05). The gluten treated with lactate showed the solubility values of 0.51 and 0.19 mg/mL at pH values 3.0 and 10.0. The gluten treated with sodium lactate showed solubility values of 0.62 and 0.21 mg/mL at pH 3.0 and 10.0.

Additionally, as shown in Figure 2B, in terms of WHC and OAC, lactylation resulted in a remarkable change compared to raw gluten: The WHC of raw gluten is 1.36 g/g. After lactylation, WHC values were reduced by 25.36 and 13.04%, respectively. However, in contrast to raw gluten, the OAC increased by 0.63 and 0.37 g in samples treated with lactate and sodium lactate (*p* < 0.05).

The emulsifying properties of gluten were investigated, and the results are displayed in Figures 2C,D. The EAI and ESI are generally used to evaluate the emulsifying properties of proteins (16). The emulsifying activities of all samples showed a U-shaped curve in which protein had the lowest EAI at pH 7.0, approximately. The EAI values of raw gluten showed as low as 16.66 m^2^/g at pH 7.0. As pH is away from the isoelectric point, the emulsifying properties gradually increase. Gluten at pH 3.0 displayed the highest EAI (102.71 m^2^/g). Meanwhile, the emulsion activities of the lactylation modified gluten sample were improved. The EAI value of lactylated glutens were 112.00 and 120.83 m^2^/g at pH 3.0, respectively. The emulsifying stabilities of all samples also showed a U-shaped curve in which protein had the lowest emulsifying stability at pH 7.0 (18.22 min). After lactylation, the gluten’s ESI were 19.48 and 21.9 min at pH 7.0, respectively.

Gluten plays an important role in the rheological properties of dough, which determines the final product quality to a certain extent. The Figures 2E,F described the relationship between the storage modulus and loss modulus of the protein sample and the oscillation frequency. Within the experiment measurement (0.1–10 Hz), the storage modulus is higher than and loss modulus in all samples. Besides, for the lactate treatment, the storage modulus and loss modulus all significantly increased. Similar results were observed in the sodium lactate treatment group. Collectively, these data suggested that lactylation significantly enhanced the viscoelasticity of the protein.

### Effects of lactylation on the secondary and tertiary structure of gluten

**FIGURE 3:**
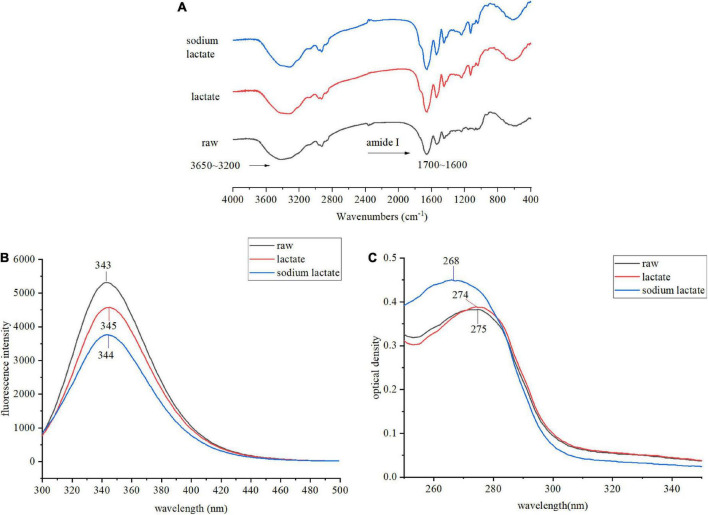
Changes in the structural properties of gluten sample. **(A)** The FTIR spectra of raw gluten, lactate treated gluten, and sodium lactate treated gluten. **(B)** The intrinsic fluorescence spectra of raw gluten, lactate treated gluten, and sodium lactate treated gluten. The excitation wavelength was 280 nm, and the emission wavelength was from 300 to 500 nm. **(C)** The UV absorption spectra of raw gluten, lactate treated gluten and sodium lactate treated gluten. The UV absorption spectra were scanned from 200 to 400 nm, while the major absorption peak spectra were from 250 to 350 nm.

The spatial structure is the basis of functional properties, so we needed to further test the effects of lactylation on the secondary and tertiary structure of gluten. The conformational structure alterations of gluten were assessed through FTIR, intrinsic fluorescence emission spectra, UV absorption spectrum, the content of SH, and SDS-PAGE. FTIR spectra of the lactate and sodium lactate-treated glutens are displayed in Figure 3A. Compared with the raw gluten, the bands’ intensity at 1850–1630 and 3650–3200 cm^–1^ were found to be increased in the lactylated gluten. The spectra in the wavenumber range of 1700–1600 cm^–1^ were analyzed by Peakfit v4.12 software to obtain the secondary structure contents (Table 1). The number of β-sheets increased significantly after lactate and sodium lactate treatment (*p* < 0.05). The number of β-sheets was 28.65% in raw gluten, and the number of β-sheets was 36.26% and 43.76% in samples treated with lactate and sodium lactate, respectively. Moreover, lactylation resulted in a significant loss of the β-turns of gluten (*p* < 0.05). The number of β-turns was 40.85% in raw gluten, and the number of β-turns was 34.99 and 27.91% in samples treated with lactate and sodium lactate, respectively. However, the lactate and sodium lactate treatment had no significant effects on the α-helices and random coils.

**TABLE 1:** The secondary structure composition of wheat gluten and lactylated wheat gluten obtained from fourier transform infrared spectroscopy (FTIR).

	β -sheet (%)	Random coil (%)	α -helix (%)	β -turn (%)
raw	28.65 ± 0.53^c^	15.23 ± 0.92^a^	15.27 ± 0.92^a^	40.85 ± 2.65^a^
lactate	36.26 ± 1.12^b^	13.87 ± 0.86^a^	14.88 ± 0.51^a^	34.99 ± 1.47^b^
sodium lactate	43.76 ± 2.18^a^	13.91 ± 0.57^a^	14.42 ± 0.47^a^	27.91 ± 1.37^c^

Data are mean ± SD (*n* = 3). Different lowercase letters in the same column mean significant differences (*p* < 0.05).

The emission fluorescence spectroscopic technique was performed to demonstrate the tertiary changes of gluten. We observed an emission spectrum with a peak position of 343 nm in the raw gluten upon excitation at 280 nm. The λmax for treated gluten samples had a shift toward longer wavelengths and the λmax were 345 and 344 nm after lactate and sodium lactate treatment, respectively. In addition, decreased maximum fluorescence intensity levels were observed after lactylation. The maximum fluorescence intensity values of lactylated glutens were significantly reduced by 13.89 and 29.13% in comparison to raw gluten.

The alteration in the tertiary structure of gluten also can be characterized by UV absorption. The UV absorbance was observed at 260–280 nm. The raw gluten exhibited maximum absorbance at 275 nm. Compared with the raw sample, the two treated samples showed a slight increase in UV absorbance and a blue shift in the position of the UV absorption peak (275–274–268 nm).

Lactylation had a marked impact on the gluten based on the assessment of secondary and tertiary structures, and some of the three-dimensional structure characteristics also could be reflected by the alterations of SH content (17). Table 2 shows the effect of lactylation on the SH content of gluten. The content of SH in raw gluten is 13.12 μM/g. After being modified with lactate and sodium lactate, the samples showed the SH values of 7.75 and 6.77 μM/g, respectively.

**TABLE 2:** Effect of lactylation on the sulfhydryl (SH) content of gluten sample.

	Content of free SH (μM/g)
raw	13.12 ± 0.75^a^
lactate	7.75 ± 0.32^b^
sodium lactate	6.77 ± 0.12^c^

Data are mean ± SD (*n* = 3). Different lowercase letters in the same column mean significant differences (*p* < 0.05).

**FIGURE 4:**
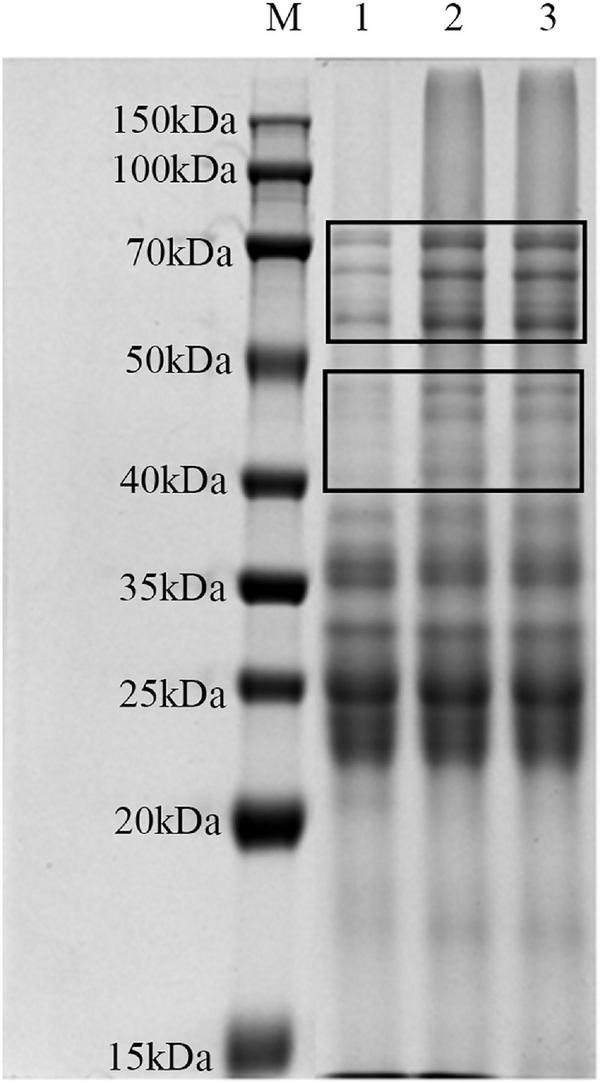
SDS-PAGE analysis of gluten exposed to different treatments: (lane M) marker; (lane 1) raw gluten; (lane 2) gluten treated by lactate; and (lane 3) gluten treated by sodium lactate.

SDS-PAGE was used for investigating the influence of lactylation on the structure of gluten (Figure 4). There were no significant differences in the number and location of the bands for all samples. The intensities of the band at 40–50 and 50–70 kDa were significantly higher than those of the raw gluten sample.

## Discussion

### Western blot analysis

Western blot is one of the important methods to detect protein with antibodies. Zhang et al. (14) showed that lactate-derived lactylation was a type of protein post-translational modification. To confirm the modification further, they developed a pan anti-Kla antibody. Subsequent studies further confirmed that lactylation played an important role in the regulation of almost all life activities by using this antibody. In this study, we first demonstrated that lactate and sodium lactate treatments could lead to lactylation of gluten. There is no lactylation in raw gluten, however, the band (15–20, 30–50 kDa) intensities of raw gluten samples were significantly strengthened treated with lactate and sodium lactate. Together, the experiment demonstrated that gluten Kla is a protein modification derived from lactate and sodium lactate. The modification sites were mainly in gliadin and LMW-GS.

### Solubility

Solubility is one of the most fundamental physical properties, which is the ratio of soluble protein content to total protein content in the protein-water system. One of the aims of protein modifications is to the enhancement solubility (18). The lowest solubility of gluten appeared at isoelectric point, because the hydrophilic and hydration repulsion force produced by charged residues were far less than hydrophobic interactions between proteins. As pH was far from the isoelectric point, the solubility gradually increased, which seemed to be ascribed to more charges on the surface of the protein molecule. In addition, Figure 2A also revealed that there has been a marked rise in the level of solubility after lactylation. This might be explained in three ways. First, this might be due to the introduction of the hydrophilic component as a result of lactylation, which enabled these groups to inform hydrogen bonds with water. Second, the newly introduced groups rendered gluten to unfold its structure, which built up the interaction force between protein’s side chains and water. This was consistent with the fluorescence spectroscopic results. Third, the hydration of protein grew bigger as a consequence of the increase of negative charge by lactylation. Research has found that phosphorylation had a similar trend, in which the introduction of phosphate resulting in the increase of negative charge benefited protein dispersion in solution (19, 20).

### Water holding capacity and oil absorption capacity

Water holding capacity (WHC) and oil absorption capacity (OAC) represent the interaction ability between protein and water/oil. On the one hand, compared with raw samples, the WHC of the treated samples had a distinct fall. After lactylation, polypeptide chains were stretched, further causing the increase in solubility. As previous mentioned, raw gluten has been a marked rise in the level of solubility after lactylation. High water-soluble protein generally showed low WHC, which might be one reason that lactylated gluten’s WHC decreased. On the other hand, OAC usually depended on the ability to embed oil. An improvement of OAC could be attributed to a decrease in protein folding degree after lactylation, and the exposed hydrophobic group further formed protein–oil complexes with oil.

### Emulsifying activity index and emulsion stability index

Desired emulsifying proteins could drop the surface tension efficiently and rapidly be absorbed into the oil-water interface, under underwent conformational change and rearrangement to form a viscoelastic cohesive film through intermolecular interactions. When deviated from the isoelectric point, the proportion of protein adsorbed at the oil-water interface increased, which improved the emulsifying properties of gluten. Protein solubility plays an important role in emulsification. Solubility is a precondition for good emulsifying properties of proteins, as only proteins with good solubility will readily diffuse into the oil-water interface. In experiments analyzing solubility, lactylated gluten solubility was better than raw gluten, hence lactylated gluten emulsification was better than raw gluten. Hu et al. (19) reported that phosphorylation with sodium trimetaphosphate could improve the solubility and emulsifying properties of rice bran protein.

### Rheological analysis

Dynamic rheological properties reflected the quality of gluten, therefore, the viscoelastic curves of gluten with different treatments were studied. All gluten samples exhibited greater storage modulus, which indicated similar solid-like behavior. Both lactate and sodium lactate treatment could significantly improve the storage modulus and loss modulus, which implied that lactylation was an effective method to improve the viscoelasticity of gluten. Raw gluten had been a marked rise in the level of solubility after lactylation, resulting in more adequate hydration of protein, and this was the major reason that made viscoelasticity improved.

### Fourier transform infrared spectroscopy analysis

The FTIR peaks at approximately 1850–1630 and 3650–3200 cm^–1^ were attributed to C=O and -OH of the protein, and the increase of the two peaks might be due to the introduction of the lactate group. The FTIR peak at approximate amide I (1600–1700 cm^–1^) reflects the secondary structure of the protein. The secondary structure comprises α-helices (1650–1660 cm^–1^), β-sheets (1610–1640 cm^–1^), β-turns (1660–1700 cm^–1^), and random coils (1640–1650 cm^–1^) (21). From the results, we observed secondary structures sensitive to lactylation. As shown in Table 1, the β-turns partially conversed to the β-sheets. First of all, the β-sheets are a relatively stable secondary structure, however, the other three secondary structures are flexible (22). The significant increase in β-sheets structure indicated that the stability of treated gluten was enhanced. Simultaneously, it was indicated that the improvement of β-sheets structures changed the protein-protein interaction and benefited the viscoelasticity of protein. This was consistent with the previous rheological results. Besides, the peptide chain alignment can be reversed by β-turns located on the protein’s surface, which enables globular proteins to be tightly compact. The significantly decreased β-turns content of the modified gluten indicated that the protein molecules were further unfolded. Zhou et al. (23) reported that frying induced the decrease of β-sheets and the increase of β-turns in gluten. The results revealed that the proteins became more extended in structure. It also has been reported a reducing pattern in the number of β-sheets and an increasing pattern in the number of β-turns, random coils, and α-helices of deamidated wheat gluten structure (24). A possible explanation is that different reagents or methods may have different effects on a protein’s secondary structure through different mechanisms.

### Intrinsic fluorescence emission spectra analysis

Several amino acids, such as Trp, can emit fluorescence. The amino acid microenvironment in the protein can be reflected by the fluorescence spectrum, and then we can speculate on the changes in tertiary structure (25). The changes in protein conformation can be measured by the changes in maximum absorption wavelength and maximum fluorescence intensity. As shown in Figure 3B, the red shift of the maximum absorption fluorescence peak indicated that amino acids’ side-chain groups are exposed to a more hydrophilic environment. Tryptophan residues were exposed to the external solution resulting from loosed protein conformation after lactylation. However, further exposed tryptophan residues did not lead to higher emissions. Such behavior could account for the decreased quantum yield. Tryptophan appears to be uniquely sensitive to quenching when tryptophan becomes exposed to an aqueous environment (26). As reported by Zhou et al. (23), protein denaturation or structural expansion would lead to increased fluorescence quenching and decreased fluorescence intensity. Meanwhile, the exposure of chromophore groups would further lead to a red shift of the maximum wavelength of protein. Sun et al. (27) also reported that some tryptophan residues were modified by reactive compounds such as ozone and hydroxyl radicals. In brief, the changes in fluorescence of gluten-induced lactylation were caused by the unfolding of protein molecules.

### UV absorption spectrum analysis

The UV spectrum of protein samples is also a common indicator to reflect the tertiary structure changes of proteins. The absorption of aromatic amino acids mainly occurs around 280 nm, including Tyr and Trp residues. As shown in Figure 3C, after lactylation, the blue shift of the maximum absorption peak of the UV spectrum indicated that the microenvironment of Tyr and Trp side-chain groups had changed (28). The increased maximum absorbance further demonstrated that lactylation had a significant effect on the gluten’s tertiary structure. As Xue et al. (29) reported, the UV absorption spectrum showed a slight blue shift and an increased maximum absorbance in the phosphorylated gliadins, and phosphorylation reduced allergic reactions to gliadins.

### Sulfhydryl analysis

Changes in gluten conformation can also be characterized by the folding of the SH (30). In our study, lactylation decreased the SH content significantly. However, it has been reported that phosphorylation increased the SH content significantly (29). There are two explanations for this phenomenon. On the one hand, the SH would be oxidized to sulfonic acid or other oxidation products during the treatment process. On the other hand, the loss of some peptides during gluten recovery might also be responsible for the decrease in SH.

### SDS-PAGE analysis

According to the changes in secondary structure and tertiary structure of gluten, the effects of these two treatments on the molecular weight distribution of gluten were further analyzed by SDS-PAGE. After lactylation, the bands of 40–70 kDa were significantly deepened, possibly because lactylation would lead to the binding of lactate groups on the gluten molecule, increasing molecular weight. Another possibility was that lactylated gluten aggregated, resulting in increased interactions between protein molecules to form aggregates.

## Conclusion

Lactylation-induced variations of gluten were systematically investigated. Compared to raw gluten, the solubility, OAC, and emulsifying property of the lactylated gluten were improved. The underlying reason could be that lactylation results in a more unfolded protein structure. The introduction of the lactate group significantly increased the viscoelasticity of gluten. In addition, the β-sheets content of gluten increased significantly, while the β-turns content was significantly reduced. Furthermore, the tertiary structure of gluten was influenced after lactylation treatment. The gluten with lactylation adopts an extended conformation instead of a folded one, resulting in the change of functional properties. Our findings indicated that lactylation, as an effective modification method, allowed gluten to be utilized for various novel wheat-related food. In the future, the modification mechanism of lactylation was further explored. In addition, more research is needed to make lactylation successfully exploit for a variety of product.

## Data availability statement

The original contributions presented in this study are included in the article/supplementary material, further inquiries can be directed to the corresponding author.

## Author contributions

YW and YL mainly analyzed the results and wrote the draft of the manuscript. MF assisted with experiments. LW and HQ contributed to the revision. All authors reviewed the manuscript.
